# Draft genome sequence data of Bifidobacterium longum strain VKPM Ac-1636, a prospective probiotic isolated from human gut

**DOI:** 10.1016/j.dib.2019.103847

**Published:** 2019-03-19

**Authors:** V.V. Zinina, A.A. Korzhenkov, A.V. Tepliuk, Yu A. Trimasova, M.V. Patrushev, I.V. Kublanov, S.V. Toshchakov

**Affiliations:** a National Research Center “Kurchatov Institute”, Moscow 123182, Russia; b Federal Institution “State Research Institute of Genetics and Selection of Industrial Microorganisms” of the National Research Center “Kurchatov Institute”, Moscow 117545, Russia

## Abstract

Data on the draft genome sequence of *Bifidobacterium longum*subsp. *longum* strain Ac-1636 is presented in this report. This strain, isolated from the digestive tract of one-year old healthy infant, was deposited in the Russian National Collection of Industrial Microorganisms as a prospective candidate for development of probiotics and probiotic foods. The 2,321,741 bp draft genome consists of 73 scaffolds with N50 of 162,253 bp. Genome annotation revealed the presence of multiple determinants of probiotic properties of this strain. The draft genome sequence data of strainAc-1636 is available in DBJ/EMBL/GenBank under the accession nos. RZHL00000000, PRJNA511803 and SAMN10644101 for Genome, Bioproject and Biosample, respectively.

**Table:** Specifications table

Subject area	Biology
More specific subject area	Microbial genomics
Type of data	Genomic sequence, predicted genes and annotation
How data was acquired	Whole genome sequencing of fragment libraries with Illumina MiSeq platform, following *de novo* genomic assembly
Data format	Annotated draft genome assembly
Experimental factors	Extraction of genomic DNA, fragment library preparation, Illumina sequencing, *de novo* assembly and annotation procedures
Experimental features	Extraction of genomic DNA was performed with standard phenol-chloroform method; fragment library was prepared with KAPA HyperPlus™ Kit; sequencing was performed with Illumina MiSeq™ system; genome assembly and annotation was performed with SPAdes assembler and RAST annotation system, respectively,
Data source location	The culture of strain VKPM Ac-1636 is deposited in Russian National Collection of Industrial Microorganisms (VKPM) in Moscow, Russian Federation. http://eng.genetika.ru/service-offer/vkpm/
Data accessibility	The draft genomesequence data of strainAc-1636 is available in DBJ/EMBL/GenBank under the accession nos. RZHL00000000 (https://www.ncbi.nlm.nih.gov/nuccore/RZHL00000000.1/), PRJNA511803 (https://www.ncbi.nlm.nih.gov/bioproject/?term=PRJNA511803) and SAMN10644101 (https://www.ncbi.nlm.nih.gov/biosample/?term=SAMN10644101) for Genome, Bioproject and Biosample, respectively.
Related research article	D.A. Sela, J. Chapman, A. Adeuya, J.H. Kim, F. Chen, T.R. Whitehead, A. Lapidus, D.S. Rokhsar, C.B. Lebrilla, J.B. German, N.P. Price, P.M. Richardson, D.A. Mills, The genome sequence of Bifidobacterium longum subsp. infantis reveals adaptations for milk utilization within the infant microbiome., Proc. Natl. Acad. Sci. U. S. A. 105 (2008) 18964–9. https://doi.org/10.1073/pnas.0809584105

**Table:** 

**Value of the data** • Initial microbiological tests revealed the capability of with this strain to inhibit the opportunistic pathogens growth. Draft genome assembly gives an opportunity to search for novel proteins and pathways of secondary metabolite biosynthesis. • Draft genome data may be used by scientific community working in the field of probiotic microorganisms to discover molecular mechanisms of probiotic production and activity. • Draft genome data may be used to broaden the knowledge on phylogenetic diversity of Bifidobacteria and, specifically, species Bifidobacterium longum

## 1 Data

In this paper we present the draft genome sequence and results of genome annotation of *Bifidobacterium longum* subsp. *longum* strain VKPM Ac-1636 obtained from the Russian National Collection of Industrial Microorganisms (RNCIM) as a potential probiotic culture. This strain, isolated in early two-thousandths from the digestive tract of one-year old healthy infant [1] was initially described as a strain of *Bifidobacterium infantis* 37b, which showed some probiotic characteristics and could be used in pediatric practice as biologically active supplement [1]. The genome was sequenced in the frame of Russian program “Genomes of industrially-relevant microorganisms” in 2018 to identify genomic determinants of its probiotic properties.

*De novo* assembly of strain VKPM Ac-1636 resulted in 73 genomic contigs of 2,321,741 bp total length. The largest contig was 274,158 bp, N50 and N90 assembly parameters were 162,253 and 33,761 bp, respectively. 60.2%GC content well correlated with GC content of other publicly available *Bifidobacterium longum* strains. Automatic *in silico* annotation with RAST pipeline [2] revealed 2049 coding sequences, 56 tRNA and at least 3 rRNA genes. Only two-third (1326) of *in silico* predicted proteins were assigned to Clusters of Orthologous Genes (COGs) [3]. The most abundant functional COG category was “Carbohydrate transport and metabolism”, comprising of more than 8% of all identified proteins. That observation well correlates with primary ecological niche of the strain, isolated from feces of healthy breastfed infant [1], and therefore targeting human milk oligosaccharides (HMO) as a valuable carbon and energy source. Second abundant functional category, “Amino acid transport and metabolism” (7.65%), accompanied with presence of genes involved in metabolism of thiamine, folate, pyridoxine and riboflavin, assumes broad prototrophy of the strain regarding amino acids and vitamins. The bile salt hydrolase gene (ELS79_06255) is involved in the resistance of the strain to the bile stress in the gastrointestinal tract of the host. Thus, draft genome sequence data of strain VKPM Ac-1636 well agree with other observations of genomic features, responsible to probiotic capabilities of different strains of genus *Bifidobacteria* [4], [5]*.*

Laboratory experiments with this strain showed that it can inhibit the growth of opportunistic pathogens (*E.coli, K. pneumoniae, S. aureus, S. faecalis, C. perfringens)* [1], but no secondary metabolite-related genes was predicted by antiSMASH server [6]. On the other hand, SMIPS server algorithm [7] predicted a gene of possible polyketide synthase type I (ELS79_09510), which might be involved in synthesis of bioactive compounds [8], and a gene for UbiA prenyltransferase (ELS79_05505) which is involved in delivery of bioactive compounds to the cell membrane [9]. However, these *in silico* observations need extensive experimental work to be reliably confirmed.(see Table 1)

**Table 1:** 

Code	Value	%age	Description
J	158	7.37	Translation, ribosomal structure and biogenesis
A	1	0.05	RNA processing and modification
K	95	4.43	Transcription
L	79	3.68	Replication, recombination and repair
B	0	0.0	Chromatin structure and dynamics
D	26	1.21	Cell cycle control, Cell division, chromosome partitioning
V	37	1.72	Defense mechanisms
T	57	2.66	Signal transduction mechanisms
M	83	3.82	Cell wall/membrane biogenesis
N	8	0.37	Cell motility
U	12	0.56	Intracellular trafficking and secretion
O	57	2.66	Posttranslational modification, protein turnover, chaperones
C	47	2.19	Energy production and conversion
G	172	8.02	Carbohydrate transport and metabolism
E	164	7.65	Amino acid transport and metabolism
F	78	3.64	Nucleotide transport and metabolism
H	63	2.94	Coenzyme transport and metabolism
I	42	1.96	Lipid transport and metabolism
P	51	2.38	Inorganic ion transport and metabolism
Q	18	0.84	Secondary metabolites biosynthesis, transport and catabolism
R	90	4.2	General function prediction only
S	60	2.8	Function unknown
X	25	1.17	Mobilome: prophages, transposons
–	723	33.71	Not in COGs

## 2 Experimental design, materials and methods

### 2.1 DNA extraction, library preparation and sequencing

Strain Ac-1636 was stored in Russian National Collection of Industrial Microorganisms as a lyophilized culture. For extraction of genomic DNA, it was re-cultivated using Blaurock medium, routinely used for cultivation of *Bifidobacterium*. Genomic DNA was extracted and purified with standard phenol-chloroform method. DNA quality and integrity were assessed by agarose gel electrophoresis as well as by measurement of A260/A280and A260/A230 by Nanodrop 1000 spectrophotometer (Thermo Fisher Scientific, USA). DNA was stored at −20 °C until further processing. DNA was fragmented using a Bioruptor™sonicator (Diagenode, Belgium) to achieve an average fragment length of 500 bp. Fragmented DNA was size-selected for fragments in range from 400 to 600 bp using agarose gel electrophoresis. Further steps of library preparation were performed with KAPA™ HyperPlus fragment library kit (Roche) according to the manufacturer's instructions. Sequencing was performed on Illumina MiSeq™ platform (Illumina, USA) using 2 × 250-cycles paired-end sequencing kit. 1,330,745 read pairs were obtained.

### 2.2 De novo assembly

Quality trimming, removal of sequencing adapters, and filtering of reads was performed with fastq-mcf [10] using the following parameters: Phred score ≥ 25, window size = 5. Overlapping paired reads were merged with the SeqPrep tool (https://github.com/jstjohn/SeqPrep/). Genome were assembled with SPAdes v 3.10 [11] in “careful” mode. To check the quality of the assembly, reads were mapped back to contigs with bowtie2 [12], mapping file was processed with samtools [13].

### 2.3 Genome annotation

Gene prediction and primary functional analysis was performed with RAST server [2]. Analysis of genes involved in the biosynthesis of secondary metabolites was made with ANTISMASH [6] and SMIPS [7] servers. COG annotation was performed as described previously [14].
